# Supplementary material for: The dependency structure of international commodity and stock markets after the Russia-Ukraine war
Source: PLoS One. 2025 Feb 6;20(2):e0316288. doi: 10.1371/journal.pone.0316288 (PMC11801530; doi:10.1371/journal.pone.0316288)
Supplement: Attachment — Submitted filename: Response to Reviewers.docx [file pone.0316288.s001.docx]

Response to reviewer

Dear Editor:

Re: PONE-D-24-41457 titled “The dependency structure of international commodity and stock markets after the Russia-Ukraine war”

We are very grateful to your and the reviewers’ critical comments and thoughtful suggestions. Based on these comments and suggestions, we have made careful modification on the original manuscript. All deletions from the text are marked in red in the revised manuscript, while additions and modifications are highlighted in blue, making them easy to identify. Some of your questions were answered below.

Once again, we acknowledge your comments and constructive suggestions very much, which are valuable in improving the quality of our manuscript.

Here are our responses to the reviewers’ comments one-by-one.

# Reviewer #1:

## **Comment1:**

The authors perform a thorough and statistically sound analysis of the dependency structure between a number of commodities and the S&P 500 index, and of its changes during different time periods. Interestingly, they compare a time of market stability with two different periods of extreme events (caused by the pandemic and by war) and find evidence of Page 5 a shift between the core of the dependence structure between stock market and commodities. The methodology of choice is well-justified by tests on the properties of the time series under study. It is also perfectly suitable for investigating the research question of the paper. The process of data cleaning, often neglected, here is presented in succinct but clear detail. The authors find interesting results, which are duly highlighted and presented clearly, and which can be useful for investors and market players at large. The only weaknesses to highlight are not methodological but cosmetic. They are the following: a. The presence of website links inside the text (as on page 3) should be avoided: consider using footnotes or references instead b. The commodities corresponding to each number in the edges of tables 5-7 and in figures 1-3 should be made explicit c. The resolution of figures 1-3 is low. If possible, it should be increased d. A few typos, slight language errors and repetitions appear. Here are some: line 61 “exogenous backgrounds”, line 63 “outbroke”, line 75 “expos-es”, line 109 “MR”, line 110 “corresponding to the corresponding”, the sentence on lines 150-152 should be made clearer, line 220 “the st”, line 288 “tree treetree1”, a period is missing after “here” and “The” should not be capitalized.

## **Response1:**

Thank you for acknowledging our thorough and statistically sound analysis of the dependency structure between commodities and the S&P 500 index across different time periods. We appreciate your recognition of our research methodology as suitable for addressing the research questions of this paper, and we are pleased to hear that the data cleaning process was clearly presented.

1. **Regarding the issue of website links in the text:**

**Response:** We have removed all website links from the main text and replaced them with footnotes to enhance the academic rigor of the paper.

2. **Concerning the clarity of commodities in tables and figures:**

**Response:** We have explicitly labeled each number corresponding to commodities in notes below Tables 5-7 and Figures 1-3, allowing readers to better understand the data. Note: 1 to 7 represent "OIL," "AU," "AG," "BEAN," "CORN," "WHEAT," and "S&P500," respectively.

3. **Regarding the resolution of figures:**

**Response:** We have enlarged the size of Figures 1-3 and improved their resolution to ensure that the information in the charts is clearly visible.

4. **Concerning typos and language errors in the text:**

**Response:** We have carefully proofread the entire manuscript and corrected all typos and language errors, including those specifically pointed out by the reviewer. We have thoroughly reviewed the text to ensure accuracy and fluency in language.

Examples of specific changes made:

• Line 61: "Exogenous backgrounds." We believe that not only was the term "exogenous backgrounds" used inappropriately, but the sentence was also unclear. The original sentence was: "However, commodity and financial market correlations based on exogenous backgrounds such as the COVID-19 outbreak and the Russia-Ukraine war remain relatively rare and have increased correlation between financial and commodity markets." It has now been changed to: "While there has been existing research on the relationship between commodity and financial markets under external shocks, studies on the deep structural changes in dependencies induced by the Russia-Ukraine war are still limited."

• Line 63: "outbroke" has been corrected to "emerged." The original sentence was: "Among the international exogenous events of recent years, COVID-19, which outbroke at the end of 2019, has become a global public health crisis, and its pandemic continues to increase economic uncertainty and cause macroeconomic depression." We have modified it to: "Baker et al. [2] highlight that in the current context of highly interconnected global financial and commodity markets, exogenous shocks such as the COVID-19 pandemic, which emerged at the end of 2019, significantly exacerbate market volatility. This outbreak not only precipitated a global public health crisis but also resulted in heightened economic uncertainty and a macroeconomic recession."

• Line 75: "expos-es". The original sentence corresponding to line 75 was: "This expos-es the possibility that the Russia-Ukraine war could affect international stocks and spill over into commodity markets, changing the existing relationship between international financial and commodity markets. Referring to studies on the impact of previous wars on financial markets." We have changed it to: "This suggests that the Russia-Ukraine war may affect international stock markets and spill over into commodity markets, thereby altering the existing relationship between international financial markets and commodity markets."

• Line 109: "MR" has been corrected to "MA". We apologize for our carelessness; this was a typographical error, and it actually refers to the "MA model," which is the moving average model.

• Line 110: "corresponding to the corresponding" has been rewritten as "corresponding to the respective variables."

• Lines 150-152: We have reconstructed this sentence to improve its clarity. The original sentence was: "On this basis, Joe (1996) proposed pair-copula construction (PPC) [21], while Bedford and Cooke (2001) proposed R-Vine copula construction based on PPC [22], which can decompose the multivariate density function into the marginal density and a series of unconditional or conditional pair-copula, thus capturing the dependence and degree of dependence between every two variables in n-dimensional variables. The R-vine copula is defined as follows:" It has now been modified to: "Building on this, Joe [16] proposed the Pair-Copula Construction (PPC). Subsequently, Bedford and Cooke [17] developed the R-Vine Copula construction based on PPC. The R-Vine Copula can decompose a multivariate density function into marginal densities and a series of unconditional or conditional pair-Copulas, thereby capturing the dependence and degree of dependence between any two variables in an n-dimensional space. The definition of the R-Vine Copula is as follows:"

• Line 220: "the st". The original sentence was: "The results of KS the st could not reject the original hypothesis, that is, each series after transformation obeyed uniform distribution and satisfied the conditions for the construction of Vine-copula." We apologize for the typographical error here and have modified this sentence to: "The results of the KS test failed to reject the null hypothesis, suggesting that the transformed series follow a uniform distribution, thereby meeting the conditions required for constructing a Vine copula model."

• Line 288: "tree treetree1" has been corrected to "Tree1". The missing period after "here" has been added, and the capitalization of "The" has also been corrected to lowercase. Specifically, the original sentence was: "Considering the space, the first level tree treetree1 is still discussed here. From Table 6, it can be seen that compared to the COVID-19 epidemic. The dependence structure of each commodity market and the stock market during the control period differs from that during the Russia-Ukraine war." It has been modified to: "Considering the length, the first-level tree, Tree 1, is still discussed here. From Table 6, it can be seen that the dependence structure during the 'no extreme events' control group differs from that during the Russia-Ukraine war."

# Reviewer #2:

## Comment1: GENERAL WRITING

Overall, the paper is poorly written, both in vocabulary (i.e., too many repetitions and ambiguous use of terms) and in the structure of the paragraphs. It is hard to understand the motivation driving this research (one can only infer it), while it should be clear in the abstract and the introduction; the methodology is hardly described and, when hinted at, it is unclear and difficult to grasp, the reader can only understand it once she gets to the "Methodology" section, whereas it should figure clearly in the introduction as well. The title suggests that the pivotal event of the analysis is the Russia-Ukraine war, however, the introduction focuses almost entirely on the COVID-19 pandemic, which is another event of major disruption for financial markets. This might create confusion upfront on what is the true goal of the research. The technical terminology is often inappropriate. For instance, the authors use coding terms that are used in R but are not suited to the text: the term "ARMA-GARCH-std", where the "std" is generally used in R to denote the Student t distribution, is not elaborated, nor is there any hint at its interpretation. Another example is the use of the term "simulation" in the context of the ARMA-GARCH, how is it a simulation when real data are used to estimate the parameters? Moreover, the terms "training group" and "control group" are not elaborated nor appropriately contextualized. Other minor inaccuracies are related to unexplained acronyms, such as AIC, BIC, KS.

**Response1:**

Thank you for your review and valuable feedback on our manuscript. We have carefully considered your comments and made the following revisions and improvements in response to the specific issues you raised.

1. **Issues with writing quality and terminology:**

**Response:** We acknowledge the shortcomings in the writing quality and use of terminology in the paper. To enhance the clarity and professionalism of the paper, we have thoroughly proofread and rewritten the entire text, particularly revising and clarifying repetitive and ambiguous terms. We have ensured that each term is clearly defined at its first occurrence and used consistently throughout the paper. As you pointed out, the term "simulation" is not appropriate for our study, and we have replaced it with more suitable terms, such as "estimated." Furthermore, we appreciate your pointing out the improper use of technical terms. We have re-examined and corrected the use of these terms to ensure their applicability and accuracy in the text. In particular, we have modified "ARMA-GARCH-std" to clarify that "std" refers to the Student's t-distribution and have made sure that all technical terms are properly contextualized and explained.

2. **Unclear research motivation:**

**Response:** We understand the importance of clear research motivation and have more explicitly articulated the driving factors of the study in the abstract and introduction. We have reorganized the introduction to more directly highlight the significance of the Russia-Ukraine war to the study while reducing the discussion of the COVID-19 pandemic to avoid confusion. Here is our revised introduction:

In recent decades, the deepening integration of global trade has provided investors in financial markets with a broader and more complex range of investment choices and portfolio strategies, while the trend of commodity financialization has also intensified ^[1]^. The link between international commodity markets and financial markets, represented by stock markets, has become increasingly closer, and the price fluctuations of assets across these markets are often interrelated. Baker et al. ^[2]^ highlight that in the current context of highly interconnected global financial and commodity markets, exogenous shocks such as the COVID-19 pandemic, which emerged at the end of 2019, significantly exacerbate market volatility. This outbreak not only precipitated a global public health crisis but also resulted in heightened economic uncertainty and a macroeconomic recession. Subsequently, the Russo-Ukrainian War, which began on February 24, 2022, introduced further substantial uncertainty into the global policy environment, exacerbating volatility in international financial markets and potentially having enduring effects on the global economy ^[3, 4]^. Policy uncertainty alters investment decisions and consumer behavior, thereby affecting financial market returns and introducing spillover effects that increase market volatility ^[5]^. Albulescu et al. ^[6]^ noted that policy uncertainty amplifies the potential for risk transmission between commodity markets and financial markets. As a result, studies investigating the relationship between commodity markets and financial markets under external shocks have emerged, covering markets such as oil, gold, stock markets, and exchange rates ^[7, 8]^. Brune et al. ^[9]^ specifically pointed out that the sudden outbreak of war increases market uncertainty, and international financial markets often react negatively to war and armed conflict events, which is reflected in falling stock prices.

The Russia-Ukraine war, as a sudden geopolitical conflict, brought new policy uncertainty to the international community ^[10]^. *The New York Times* reported that on the day the war broke out, the S&P 500 index fell by more than 10%^[11]^. This suggests that the Russia-Ukraine war may affect international stock markets and spill over into commodity markets, thereby altering the existing relationship between international financial markets and commodity markets. The Russia-Ukraine war not only increased the uncertainty surrounding business operations but also had a significant negative impact on global stock market returns. Although the war occurred primarily between Russia and Ukraine, economic globalization magnified its impact, causing ripple effects on the neighboring countries and the UN member states calling for a ceasefire^[10]^. In the commodity market, the war is expected to have a particularly pronounced impact due to Russia's key role in energy supply and Ukraine's importance in agricultural supply, with oil being a critical commodity in international markets ^[12]^.

With the deepening of global economic integration, the interdependence between international financial markets and commodity markets has grown, meaning that price fluctuations and risks in one asset market may spill over to other markets. Analyzing market dependencies during major external shocks helps investors better allocate assets. While there has been existing research on the relationship between commodity and financial markets under external shocks, studies on the deep structural changes in dependencies induced by the Russia-Ukraine war are still limited. This study aims to analyze the relationship between international commodity markets and stock markets under the external shock of the Russia-Ukraine war. We selected the prices of six commodity futures (oil, gold, silver, soybean, corn, and wheat) and the S&P 500 index, using ARMA-GARCH models to fit the marginal distributions of each market and employing the R-Vine Copula structure to analyze the dependencies between these marginal distributions. To ensure the robustness of the data, we excluded irregular trading days and constructed independent R-Vine Copula structures for the periods of the Russia-Ukraine war, the pre-war COVID-19 pandemic, and periods without major external shocks. This analysis aims to better understand the impact of the Russia-Ukraine war on the dependence structure between commodity and stock markets.

By comparing the dependency structures between commodity and stock markets before and after the war, as well as during the pandemic, we found that the Russia-Ukraine war significantly strengthened the dependence between commodity and stock markets, with oil emerging as a core linked commodity, showing a pronounced transmission effect. Additionally, the inverse relationship between the stock market and precious metals indicates that investors tend to treat precious metals as safe-haven assets in high-risk environments. Furthermore, the price fluctuations of agricultural commodities during the Russia-Ukraine war showed stronger correlations with the energy market, further demonstrating the heightened spillover effects between markets under the war context. These findings align with our initial expectations.

The remainder of the paper is structured as follows: Section 2 introduces the ARMA-GARCH model and R-Vine Copula method; Section 3 describes the data sources and sample characteristics, providing descriptive statistics; Section 4 presents the empirical analysis results, exploring the differences in market dependencies under different backgrounds; Section 5 concludes the paper, discussing the implications of these findings for portfolio design and risk management.

**3. Issues with unclear method description:**

**Response:** We recognize that the description of the methods section was not clear enough. Therefore, we have introduced the research methods earlier in the introduction, emphasizing the following methods in the **Introduction**:

We selected the prices of six commodity futures (oil, gold, silver, soybean, corn, and wheat) and the S&P 500 index, using ARMA-GARCH models to fit the marginal distributions of each market and employing the R-Vine Copula structure to analyze the dependencies between these marginal distributions. To ensure the robustness of the data, we excluded irregular trading days and constructed independent R-Vine Copula structures for the periods of the Russia-Ukraine war, the pre-war COVID-19 pandemic, and periods without major external shocks. This analysis aims to better understand the impact of the Russia-Ukraine war on the dependence structure between commodity and stock markets.

**4. Issue with unexplained acronyms:**

**Response:** We acknowledge that unexplained acronyms may cause confusion for readers. We have provided concise definitions and explanations for all acronyms at their first occurrence in the text, such as AIC, BIC, and KS.

For example, the explanation for AIC, BIC, and HQ is as follows:

Among them, AIC (Akaike Information Criterion), BIC (Bayesian Information Criterion), and HQ (Hannan-Quinn Information Criterion) are standards used for model selection, helping to determine the optimal model specification. Both AIC and BIC balance the model's fit and complexity; AIC tends to select models with higher complexity, while BIC favors more parsimonious models. The HQ criterion provides a compromise between AIC and BIC. Generally, smaller AIC, BIC, and HQ values indicate that the model has a better fit and lower complexity.

Regarding the explanation for KS:

Finally, following the marginal distribution fitting, we applied probability integral transformations to convert the distributions into standardized residuals, followed by the Kolmogorov-Smirnov (KS) test. The results of the KS test failed to reject the null hypothesis, suggesting that the transformed series follow a uniform distribution, thereby meeting the conditions required for constructing a Vine copula model.

## Comment2: BIBLIOGRAPHY

Overall, the bibliography is non-exhaustive and the cited references are mostly either unrelated to the topic of discussion or missing where needed (e.g., line 88 in the manuscript). In the text, the citing system is not uniform, sometimes the cited papers appear by full name and year and other times by the reference number in square brackets in the "References" section.

### Response2:

**1. Issue with the comprehensiveness of references:**

**Response:** We acknowledge the shortcomings in the references section, particularly in terms of relevance and completeness. To enhance the academic depth and support of our paper, we have conducted an extensive literature review and ensured that all cited literature effectively supports our research arguments.

**2. Issue with the relevance of cited references:**

**Response:** We have carefully examined every citation in the paper to ensure that all referenced literature is directly related to our research topic. For the specific line you pointed out (for example, line 88 in the manuscript), we have added appropriate references to provide more substantial literary support.

**3. Issue with the consistency of the citation system:**

**Response:** We recognize that inconsistencies in citation formats may affect the reader's experience. Therefore, we have standardized the citation format throughout the paper, ensuring that all citations follow the same standard, i.e., using reference numbers in brackets in the main text and listing complete reference information in the "References" section.

## Comments 3: EMPIRICAL ANALYSIS

While the use of R-vine copulas to address changes in the dependence structure is new in the context of the Russia-Ukraine war, the empirical analysis in the ARMA-GARCH estimation is not precise in its description.

1. The data source is not indicated.

2. What justifies the use of different GARCH models? Is there an interest in capturing asymmetry? Was this tested (e.g., using a negative size bias test or similar)? Also, neither of the models is explained.

3. The AIC criterion alone is not sufficient for model selection, but Table 3 and Table 4 do not report any other additional selection criteria, e.g., BIC, Hannan-Quinn (HQ).

4. At the beginning of the empirical analysis, the estimation seems to be performed under the assumption of Gaussian distribution, but then the parameters in Table 4 include the shape coefficient, which is typically related to the Student t; similarly, the caption of the same table reads "ARMA-GARCH-std." It is inferred that it is a consequence of the results of the Jarque-Bera test for normality, but this step needs further elaboration.

5. Table 3 does not report the significance of the estimated coefficients.

### Response3:

1. **Issue with unspecified data sources:**

**Response:** We recognize that failing to specify the data sources in the original manuscript was an oversight. To address this, we have revised the methods section to include detailed information about the data sources, such as the institutions providing the data, the specific content of the data, and the time periods covered. We apologize for any inconvenience this may have caused and appreciate your understanding and patience. The specific changes can be found in the first paragraph of the DATA section:

The data for this study is sourced from the Nanhua Commodity Index Database, covering key financial indicators: WTI crude oil futures settlement prices, the closing prices of gold on the London International Financial Futures Exchange (LIFFE), closing prices of silver in London, and closing prices of soybean, corn, and wheat futures on the Chicago Board of Trade (CBOT), along with the S&P 500 index. The research period is specifically chosen from February 24th, 2022, to June 1st, 2022, corresponding to the Russia-Ukraine conflict period. For the control group, data from December 1st, 2019, to December 31st, 2020, is selected to represent the COVID-19 period, and data from January 1st, 2014, to December 31st, 2017, is selected as the sample for a period without extreme events. In this paper, we meticulously filtered the sample data, removing non-synchronous trading dates and excluding records with missing values. Applying Equation (8) to the seven data sets, we derived the daily return series, which exhibit temporal smoothness and are regarded as first-order differenced series.

2. **Issue with the rationale for using different GARCH models and testing:**

**Response:** Thank you for pointing out the lack of explanation regarding our model selection. We have provided a detailed explanation in the methods section for choosing different GARCH models, and the reasons for using different types are as follows:

Firstly, after conducting the Jarque-Bera test, we have identified the non-normal distribution characteristics of some series. For series that are normally distributed, we select the Normal Distribution; for those that are not normally distributed, we choose the Student's t-Distribution or the Generalized Error Distribution.

Secondly, for a range of GARCH models, including sGARCH, eGARCH, tGARCH, GJRGARCH, and APARCH, we initially select the GARCH model based on the principle of minimizing the AIC and then perform a KS test after selection. If the KS test result is significant, it indicates a high degree of fit, and we retain the model. If the KS test result is not significant, indicating a low degree of fit, we further select the combination with the minimum BIC or HQ and perform the KS test on the residuals again to determine the appropriate GARCH model. The modifications in the paper are as follows:

Firstly, to address the issues of autocorrelation and heteroskedasticity in time series data, we have employed the ARMA-GARCH modeling framework. Specifically, we have fitted an ARMA(m, n)-GARCH(1,1) model to each series. The optimal orders of the ARMA model, denoted by m and n, were determined using the auto.arima function in R, which selects the best orders based on the Akaike Information Criterion (AIC) and the Bayesian Information Criterion (BIC). Following the selection of the optimal lag orders, we conducted parameter estimation, and the results are presented in Table 3. And Table 3 includes the estimated parameters for both the ARMA and GARCH components, and we assessed the model fit by examining the significance of the coefficients and the residuals. In this way, we ensure that the model accurately captures the dynamic characteristics of each series.

Next, in selecting the type of GARCH model, we considered sGARCH, APARCH, and GJR-GARCH models. The optimal model was chosen using the AIC, BIC, and HQ criteria to achieve a balance between model fit and complexity, thereby avoiding overfitting. After fitting the model, parameter estimation was conducted, as presented in Table 4, where most parameters are statistically significant.

Then, regarding the choice of distribution, we base our selection on the Jarque-Bera (J-B) test results in Table 2, which indicate whether the data conforms to a normal distribution. For series with non-significant J-B test results, we use the normal distribution (norm); for those significantly deviating from normality, we select the Student’s t-distribution (std) or the Generalized Error Distribution (ged). The normal distribution is appropriate for data exhibiting relatively symmetric returns with no substantial skewness. In contrast, the t-distribution suits series with fat tails, commonly observed in financial data, while the ged is preferable when there is evidence of high kurtosis or extreme values, as it better accommodates heavy tails than the normal distribution. These distribution choices are based on the specific characteristics of each series to ensure accurate representation of data properties.

Finally, following the marginal distribution fitting, we applied probability integral transformations to convert the distributions into standardized residuals, followed by the Kolmogorov-Smirnov (KS) test. The results of the KS test failed to reject the null hypothesis, suggesting that the transformed series follow a uniform distribution, thereby meeting the conditions required for constructing a Vine copula model. Consequently, the ARMA-GARCH model provides a robust foundation for constructing the Vine copula dependency structure. Due to length limitations, detailed KS test results are omitted.

3. **Issue with the contradiction between Gaussian distribution assumption and Student's t-distribution:**

**Response:** We recognize the confusion regarding the distribution assumptions in our description. We have detailed in the methods section why we shifted from the Gaussian distribution to using the Student's t-distribution, including the results of the Jarque-Bera normality test and its impact on model selection. Some of the series in our study follow a Gaussian distribution (normal distribution), for which we have chosen the ARMA(m, n)-GARCH(1,1)-norm (normal distribution ARMA-GARCH) combination. For series that do not follow a normal distribution, we then select the use of the Student's t-distribution (std) or the Generalized Error Distribution (ged). In the paper, we explain as follows:

Then, regarding the choice of distribution, we base our selection on the Jarque-Bera (J-B) test results in Table 2, which indicate whether the data conforms to a normal distribution. For series with non-significant J-B test results, we use the normal distribution (norm); for those significantly deviating from normality, we select the Student’s t-distribution (std) or the Generalized Error Distribution (ged). The normal distribution is appropriate for data exhibiting relatively symmetric returns with no substantial skewness. In contrast, the t-distribution suits series with fat tails, commonly observed in financial data, while the ged is preferable when there is evidence of high kurtosis or extreme values, as it better accommodates heavy tails than the normal distribution. These distribution choices are based on the specific characteristics of each series to ensure accurate representation of data properties.

4. **Issue with not reporting the significance of estimated coefficients:**

**Response:** Thank you for pointing out that we did not report the significance of the estimated coefficients. We have now included the significance levels of all estimated coefficients in Table 3 to better assist readers in understanding the model results. The revised Table 3 is as follows:

**Table 3. ARMA parameter estimation**

| **Parameters** | **Estimation of ARIMA parameters during the Russia-Ukraine war** | | | | | | |
| --- | --- | --- | --- | --- | --- | --- | --- |
|  | **OIL** | **AU** | **AG** | **BEAN** | **CORN** | **WHEAT** | **S&P500** |
| ar(1) | -0.691^***^ | -0.928^***^ | -0.943^***^ | -0.836^***^ | -0.304^**^ | -0.537^***^ | -0.923^***^ |
| ar(2) | -0.528^***^ | -0.678^***^ | -0.621^***^ | -0.822^***^ |  |  | -0.718^***^ |
| ar(3) | -0.394^***^ | -0.427^***^ | -0.246^**^ | -0.437^***^ |  |  | -0.564^***^ |
| ar(4) |  | -0.202^**^ |  | -0.289^**^ |  |  | -0.204^*^ |
| ar(5) |  |  |  |  |  |  |  |
| ma(1) |  |  |  |  | -1.000^***^ |  |  |
| ma(2) |  |  |  |  |  |  |  |
| intercept | 0.000 | 0.000 | 0.001 | 0.000 | 0.000 | 0.000 | 0.000 |
| AIC | -222.299 | -391.098 | -342.646 | -350.204 | -336.676 | -229.126 | -335.615 |
| BIC | -211.276 | -377.870 | -331.623 | -336.976 | -327.857 | -222.512 | -322.387 |
| HQ | -6.147 | -8.656 | -7.945 | -8.049 | -7.926 | -6.267 | -7.830 |
| LL | 116.150 | 201.549 | 176.323 | 181.102 | 172.338 | 117.563 | 173.808 |
| **Parameters** | **Estimation of ARIMA parameters during COVID-19** | | | | | | |
|  | **OIL** | **AU** | **AG** | **BEAN** | **CORN** | **WHEAT** | **S&P500** |
| ar(1) | -0.933^***^ | -0.610^***^ | -0.484^***^ |  | 0.051 | -0.552 | -0.718^***^ |
| ar(2) | -0.885^***^ | -0.268^***^ |  |  | -0.067 |  |  |
| ar(3) | -0.789^***^ |  |  |  | 0.032 |  |  |
| ar(4) | -0.391^***^ |  |  |  | -0.107 |  |  |
| ar(5) | -0.160^***^ |  |  |  |  |  |  |
| ma(1) |  |  |  | -0.855^***^ | -1.000 |  |  |
| ma(2) |  |  |  | -0.145^**^ |  |  |  |
| intercept | 0.003 | 0.001 | 0.001 | -0.002^**^ | -0.003 | -0.014 | 0.000 |
| AIC | 1818.344 | 921.556 | 1282.048 | 752.220 | 906.978 | 2238.286 | 2127.089 |
| BIC | 1843.507 | 935.935 | 1292.832 | 766.599 | 932.141 | 2251.053 | 2139.856 |
| HQ | 3.941 | 0.595 | 1.930 | -0.053 | 0.537 | 1.460 | 1.246 |
| LL | -902.170 | -456.780 | -638.020 | -372.110 | -446.490 | -553.960 | -586.470 |

Note: *, **, *** indicate significant at the 10%, 5%, and 1% levels

5. **Inadequate criteria for model selection:**

**Response:** We agree that using only the Akaike Information Criterion (AIC) as a model selection criterion is insufficient. Therefore, we have supplemented other model selection criteria, such as the Bayesian Information Criterion (BIC) and the Hannan-Quinn (HQ) criterion, in the methods section and in Table 3 and Table 4 to provide a more comprehensive model comparison. The revised Table 3 can be seen above, and the revised Table 4 is as follows:

**Table 4. ARMA-GARCH Parameter estimation**

| **During the Russia-Ukraine War** | | | | | | | |
| --- | --- | --- | --- | --- | --- | --- | --- |
|  | **OIL** | **AU** | **AG** | **BEAN** | **CORN** | **WHEAT** | **S&P500** |
| GARCH model | GJRGARCH (1,1)-norm | APARCH (1,1)-std | sGARCH (1,1)-norm | sGARCH (1,1)-std | apARCH (1,1)-ged | sGARCH (1,1)-std | sGARCH (1,1)-norm |
| $\mu$ | 0.001^***^ | 0.000 | 0.000 | 0.000 | 0.001 | 0.000 | 0.000 |
|  | 0.000 | 0.016 | 0.001 | 0.001 | 0.001 | 0.002 | 0.001 |
| $\omega$ | 0.000 | 0.000 | 0.000 | 0.000 | 0.000 | 0.000^**^ | 0.000 |
|  | 0.000 | 0.000 | 0.000 | 0.001 | 0.000 | 0.000 | 0.000 |
| $\alpha$ | 0.056^***^ | 0.021 | 0.000 | 0.000 | 0.371 | 0.095 | 0.095 |
|  | 0.000 | 18.525 | 0.001 | 0.035 | 0.286 | 0.084 | 0.094 |
| $\beta$ | 1.000^***^ | 0.806 | 0.994^***^ | 0.000 | 0.204 | 0.832^***^ | 0.831^***^ |
|  | 0.000 | 36.993 | 0.003 | 0.823 | 0.501 | 0.062 | 0.066 |
| $\gamma$ | -0.134^***^ | 0.555 |  |  | -0.124 |  |  |
|  | 0.001 | 550.240 |  |  | 0.267 |  |  |
| $\delta$ |  | 3.302 |  |  | 3.500^***^ |  |  |
|  |  | 63.713 |  |  | 0.242 |  |  |
| shape |  | 11.679 |  | 6.633^*^ | 2.369*** | 7.767 |  |
|  |  | 163.298 |  | 3.991 | 0.722 | 5.403 |  |
| LL | 120.279 | 203.576 | 176.698 | 179.693 | 163.492 | 122.480 | 173.017 |
| **During the COVID-19** | | | | | | | |
| GARCH model | **OIL** | **AU** | **AG** | **BEAN** | **CORN** | **WHEAT** | **S&P500** |
|  | GJRGARCH (1,1)-ged | GJRGARCH (1,1)-std | sGARCH (1,1)-std | APARCH (1,1)-std | sGARCH (1,1)-std | sGARCH (1,1)-std | GJRGARCH (1,1)-ged |
| $\mu$ | 0.035^***^ | -0.029 | -0.054 | -0.003 | -0.001^***^ | -0.020 | 0.032^*^ |
|  | 0.002 | 0.025 | 0.046 | 0.000 | 0.001 | 0.036 | 0.019 |
| $\omega$ | 0.507^***^ | 0.119 | 0.865 | 0.002 | 0.053^***^ | 0.009 | 0.101^***^ |
|  | 0.237 | 0.074 | 0.747 | 0.056 | 0.012 | 0.026 | 0.050 |
| $\alpha$ | 0.426^***^ | 0.149^**^ | 0.211 ^***^ | 0.000 | 0.000 | 0.004 | 0.474^***^ |
|  | 0.116 | 0.069 | 0.092 | 0.068 | 0.000 | 0.008 | 0.126 |
| $\beta$ | 0.744^***^ | 0.744^***^ | 0.736^***^ | 0.999^***^ | 0.972^***^ | 0.995^***^ | 0.700^***^ |
|  | 0.052 | 0.095 | 0.129 | 0.003 | 0.005 | 0.001 | 0.055 |
| $\gamma$ | -0.342^***^ | 0.151 |  | -1.000 |  |  | -0.351^**^ |
|  | 0.132 | 0.197 |  | 0.081 |  |  | 0.144 |
| $\delta$ |  |  |  | 1.286 ^**^ |  |  |  |
|  |  |  |  | 0.591 |  |  |  |
| shape | 0.974^***^ | 5.939^***^ | 3.981 ^***^ | 7.288 | 4.204 ^***^ | 11.964 | 1.414^***^ |
|  | 0.135 | 2.064 | 1.218 | 19.958 | 1.283 | 9.552 | 0.163 |
| LL | -716.9853 | -430.634 | -616.4662 | -356.599 | -438.287 | -551.850 | -483.634 |

Note: The table reports Robust Standard Errors. *, **, *** indicate significant at the 10%, 5%, and 1% levels；

## Comments 4. OTHER REMARKS

The results are poorly discussed. There is barely any critical thinking behind the comments on the results and there are no considerations of the systemic implications of the analysis. How are the results informative for the reader and how do they answer the research question? This is not clear enough and ambiguous. Overall, the idea is valid, and the methodology per se is sound but the execution is chaotic and often inaccurate, or at least inaccurately explained.

### Response4:

We acknowledge that the discussion of the results in the original manuscript was not deep enough and lacked critical analysis. To address this issue, we have added an in-depth analysis of the results, including critical thinking about the outcomes and discussions on their systemic implications. Furthermore, in the conclusion section, we have included policy recommendations based on the results. Below is our revised conclusion with the new text highlighted in blue:

In this paper, we use ARMA-GARCH-std to construct a Vine Copula model to study the dependence structure of international commodity and stock markets during the Russia-Ukraine war. In the sample selection, we select seven sets of series, namely, WTI crude oil futures settlement price OIL, London gold closing price AU, London silver closing price AG, CBOT soybean futures closing price BEAN, CBOT corn futures closing price CORN, CBOT wheat futures closing price WHEAT, and S&P 500 index S&P500. We set the sample period from 24^th^ February 2022 to 1^st^ June 2022 based on the period of the Russia-Ukraine war. After removing the treatment of non-common trading day samples, this paper derives seven sets of differential one-period daily return series for the two sample periods.

We select the optimal order of ARMA based on the above 7 sets of time series according to the AIC information criterion and construct ARMA(m,n)-GARCH(1,1) models as their marginal distribution functions for each series, respectively, where m and n are the optimal lagged orders of ARMA, and the GARCH types include sGARCH, eGARCH, and GJR-GARCH. By the KS test, the fitted standard residuals of each time series satisfy the condition of uniform distribution, and the vine copula can be constructed. We construct the R-Vine copula structure on this basis and derive the research results of the phase-dependent structure. As a comparison, this paper selects samples during the COVID-19 pandemic and periods without extreme events, and the vine copula structure is also constructed.

Through the empirical study, the main findings of this paper are as follows. During the Russia-Ukraine war, the structure of the vine connecting each market was still R-vine, but observing its structure and correlation coefficient Kendall's tau shows that during this period, the crude oil futures OIL serves as a connecting hub between the markets, and the OIL is directly linked to the soybean market BEAN and corn market CORN, representing agricultural commodities, as well as the silver market AG for precious metals. This suggests that price volatility in the crude oil market may be contagious in the same direction as in the agricultural and precious metals markets. A look at the correlation coefficient tau shows that the stock market S&P500 and the gold market AU are negatively correlated, which indicates the possibility of reverse price volatility transmission between these two markets, further suggesting that asset allocation to the equity market and the gold market may play a hedging role for asset risk. In addition, looking at the correlation coefficients between the rest of the markets, we can see that the rest of the tau values are positive and increased compared to the period of the COVID-19 epidemic, and the markets are more closely correlated. Overall, the vine copula structure during the Russia-Ukraine war shows, first of all, that crude oil became the key commodity behind the war. This finding also further reveals the importance of crude oil in the war. The war has increased policy uncertainty in the international community, which has contributed to price volatility risks in commodity and stock markets, and Russia's energy supply contraction has further exacerbated the propagation of crude-driven price volatility risks in international markets. Secondly, the war between the two countries affected the international supply of agricultural products. Energy price volatility is contagious to international agricultural markets, and Ukraine, an important exporter of agricultural products, has seen a contraction in production and exports of all types of agricultural products. The war could not only further amplify the spread of price risks in financial and commodity markets but also exacerbate the international food security problems that already existed during COVID-19. Lastly, crude oil price fluctuations were also homogeneously transmitted to the precious metals market, while allocating assets in the gold and stock markets had the potential to diversify asset risk.

Finally, to further observe changes in the dependency structure, we introduce the pre-war COVID-19 epidemic sample and the sample without extreme events. During the COVID-19 epidemic, the vine structure connecting each market is the R-vine structure and observing tau rank correlation coefficients and tree1 plots for tree1 between each market shows that the corn futures market is the hub connecting the other commodity markets and the stock market. The correlation coefficients between each market are positive, with mostly smaller tau values than during the Russia-Ukraine war, indicating the possibility of homogeneous transmission of price fluctuations across markets but with less dependence than during the Russia-Ukraine war. The largest tau values are found between the gold AU and silver AG markets and between the soybean BEAN and corn CORN markets, i.e., the dependence is strong, and the possibility of homogeneous transmission of fluctuations is high. This suggests that food security issues became a central international concern during the COVID-19 epidemic and that volatility in agricultural prices may be transmitted homogeneously to other commodities and stock markets. While the structure of vine copula in the comparison period without extreme events is a chain-like D-vine, and although it does not have a market with the presence of a key pivotal position like the vine structure during the Russia-Ukraine war, the gold (AU), silver (AG), and soybean (BEAN) markets are more correlated with other markets in the comparison period, precious metals and agricultural products dominate the overall structure. In addition, tau values across markets during periods without extreme events are generally lower than tau values during the Russia-Ukraine war, with lower dependence. This further suggests that the epidemic and war shocks have enhanced the interdependence of international commodity and equity markets and that the financial markets are increasingly linked to each other. Overall, based on the comparative results, we find that the Russia-Ukraine war has increased the dependence on the commodity and stock markets, and the core of the dependence structure has shifted from agricultural products and precious metals to crude oil.

Overall, based on our comparative analysis, we find that the Russia-Ukraine war has intensified the interdependence between commodity and stock markets, shifting the core dependency structure from agricultural and precious metals markets to the oil market. In summary, extreme events like the Russia-Ukraine war and the COVID-19 pandemic have significantly heightened international market interdependence and volatility risks through distinct pathways. This study reveals the evolving structure of contagion chains between markets, especially highlighting the central role of key hub markets in the transmission of shocks. These findings offer a theoretical foundation and practical insights for policy-making, risk management, and the stabilization of global markets. Based on our findings, we propose the following policy recommendations.

First, as oil price fluctuations during wartime significantly transmit to agricultural and precious metals markets, policymakers may consider stabilizing energy prices to reduce spillover effects on other markets. For instance, dynamic price adjustments in the energy market during extreme events can mitigate widespread market contagion driven by oil price volatility. Additionally, implementing market volatility control mechanisms, such as price limit systems and enhanced market transparency, can alleviate contagion effects during such events. Regulatory authorities should closely monitor key markets, such as oil and agricultural commodities, and employ necessary price adjustments to contain the spread of systemic financial risks.

Second, our research highlights a negative correlation between stock and gold markets during wartime, offering a valuable reference for asset allocation strategies. During periods of high volatility, strategically including assets like gold and stocks, which display inverse volatility characteristics, can effectively diversify risks and strengthen portfolio resilience. In extreme event environments, investors should pay closer attention to shifts in dependency structures to optimize portfolio diversification.

Finally, extreme events have substantially increased the interdependence between international markets, with particularly strong contagion effects among commodity markets during the Russia-Ukraine war. To address these volatility risks, nations should reinforce international cooperation, enhance supply chain management, and stabilize global trade agreements, particularly in critical resources such as energy and food. Establishing a global energy reserve system to mitigate disruptions in energy supply due to events like war is essential. Furthermore, the international community could strengthen food reserves and supply chain resilience to reduce the impact of extreme events on agricultural market volatility, thereby safeguarding global food security and market stability.

These policy recommendations, derived from our research, provide a comprehensive approach to managing market risks and adjusting dependency structures dynamically in the face of extreme events, aiding both policymakers and investors in making more informed decisions.

# Others

We have also made several other improvements throughout the manuscript to enhance clarity and readability. These include:

1. The **References** in the full text have been updated, so the contents and serial numbers of the references have been changed.

2. In order to standardize the narrative, we have replaced SP500 with S&P500 in the full text; R-vine with R-Vine in the full text; and apARCH with APARCH and gjrGARCH with GJR-GARCH in the main text.

We hope that the revised manuscript meets the reviewers’ and your expectations. We believe that the changes have significantly improved the quality of our work. Thank you once again for your time and consideration.

Sincerely,
